# Evaluation of minimally invasive sampling methods for detecting *Avipoxvirus*: Hummingbirds as a case example

**DOI:** 10.3389/fvets.2022.924854

**Published:** 2022-08-24

**Authors:** Aoife N. Galvin, Pranav S. Pandit, Simon G. English, Rachel C. Quock, Ruta R. Bandivadekar, Rita R. Colwell, Barbara W. Robinson, Holly B. Ernest, Mollie H. Brown, Ravinder N. M. Sehgal, Lisa A. Tell

**Affiliations:** ^1^Department of Biology, San Francisco State University, San Francisco, CA, United States; ^2^EpiCenter for Disease Dynamics, One Health Insititute, School of Veterinary Medicine, University of California, Davis, Davis, CA, United States; ^3^Department of Medicine and Epidemiology, School of Veterinary Medicine, University of California, Davis, Davis, CA, United States; ^4^Hummingbird Monitoring Network, Patagonia, AZ, United States; ^5^Wildlife Genomics and Disease Ecology Laboratory, Department of Veterinary Sciences, University of Wyoming, Laramie, WY, United States

**Keywords:** avian pox, virus, *Trochilidae*, disease ecology, prevalence

## Abstract

Avian pox is a common avian virus that in its cutaneous form can cause characteristic lesions on a bird's dermal surfaces. Detection of avian pox in free-ranging birds historically relied on observations of visual lesions and/or histopathology, both which can underestimate avian pox prevalence. We compared traditional visual observation methods for avian pox with molecular methods that utilize minimally invasive samples (blood, toenail clipping, feathers, and dermal swabs) in an ecologically important group of birds, hummingbirds. Specifically, avian pox prevalence in several species of hummingbirds were examined across multiple locations using three different methods: (1) visual inspection of hummingbirds for pox-like lesions from a long-term banding data set, (2) qPCR assay of samples from hummingbird carcasses from wildlife rehabilitation centers, and (3) qPCR assay of samples from live-caught hummingbirds. A stark difference in prevalences among these three methods was identified, with an avian pox prevalence of 1.5% from banding data, 20.4% from hummingbird carcasses, and 32.5% from live-caught hummingbirds in California. This difference in detection rates underlines the necessity of a molecular method to survey for avian pox, and this study establishes one such method that could be applied to other wild bird species. Across all three methods, Anna's hummingbirds harbored significantly higher avian pox prevalence than other species examined, as did males compared with females and birds caught in Southern California compared with Northern California. After hatch-year hummingbirds also harbored higher avian pox prevalences than hatch-year hummingbirds in the California banding data set and the carcass data set. This is the first study to estimate the prevalence of avian pox in hummingbirds and address the ecology of this hummingbird-specific strain of avian pox virus, providing vital information to inform future studies on this charismatic and ecologically important group of birds.

## Introduction

Diseases and pathogens are known to have devastating effects on wild bird populations, and climate change and land use change can exacerbate these issues, increasing disease risk and exposure (1, 2). Monitoring of avian diseases is a crucial first step in addressing the impact of these diseases in bird populations but is difficult to implement. Avian pox is a widespread avian disease that infects over 230 species of birds globally, accounting for over 3% of overall bird diversity (3, 4). Avian pox infections are caused by double-stranded DNA viruses from the genus *Avipoxvirus*., which target epithelial tissues and develop in the cytoplasm of epithelial cells, producing cytoplasmic inclusion bodies (3, 4). Avian pox is commonly transmitted by arthropod mechanical vectors, such as mosquitoes, and can also be spread through direct contact with infected birds or infected surfaces, such as bird feeders (5, 6). Infected birds develop characteristic lesions on dermal surfaces, and in the more severe diphtheritic form, inflammation of internal mucous membranes (7). Avian pox infections can cause high mortality rates in captive bird populations, but the impacts on wild birds are harder to ascertain due to difficulties in recapturing infected birds and estimating mortality in wild populations (7–11). Severity of avian pox infections appear to vary between species, and infections have been shown to change bird behavior, making birds more susceptible to secondary infections and predation, decrease reproductive success, and decrease survival rates (11–13). Despite numerous studies on avian pox in various bird species, a wide-spread method that accurately estimates prevalence of avian pox in wild bird populations without invasively sampling birds has not been well implemented.

Prevalence of avian pox varies widely in bird species. Areas with a long co-evolutionary history between avian pox and host report avian pox prevalences from 0.5 to 12%, while areas where avian pox introductions are believed to be more recent, e.g.: remote islands groups like Hawaii, etc., report avian pox prevalences of up to 86% (14). Multiple methods exist currently for estimating avian pox infections in wild bird populations. The most used method is visual inspection of birds for presence of pox-like lesions, which can be problematic for multiple reasons. Pox-like lesions alone are not a definitive diagnosis of avian pox infection and need to be coupled with histopathology of lesions to identify Bollinger bodies to confirm the lesion was caused by avian pox.

Additionally, pox-like lesions can be small and easily missed, especially if scientists handling the birds are not specifically looking for lesions. Lesions also appear on birds during peak of infection, and so birds recently infected or recently recovering can be missed in these prevalence counts. Birds could also be infected and possibly not develop pox-like lesions, leading to these visual counts underestimating pox prevalence. More recently, molecular methods have been used to identify avian pox infection status of birds by using PCR to amplify avian pox virus sequences. Although more accurate than visually inspecting birds, molecular methods require more work and have yet to be used on a wide scale. The best sample type to take from birds to use for molecular methods has also not been standardized, and a wide array of blood and integument samples have been used to assess avian pox prevalence (15–17).

Avian pox is known to infect hummingbirds, but the origin of avian pox in the *Trochilidae* family and the longevity of the co-evolutionary relationship between hummingbirds and their avian pox strain is unknown. The first report of a hummingbird with pox-like symptoms was in 1958 (5), and since then only three other studies have documented avian pox in hummingbirds (18–20). All studies have been on Anna's Hummingbirds (*Calypte anna*) caught or found in California, although field observations have noted pox-like lesions on other sympatric species of hummingbirds in California, such as Allen's Hummingbirds (*Selasphorus sasin*) and Black-chinned Hummingbirds (*Archilochus alexandri*) (Tell, personal communication). Godoy, et al. sequenced the 4b core protein gene of the avian pox strain from lesions of infected Anna's Hummingbirds and found that it formed a unique sub-clade in relation to previously sequenced avian pox strains (20). Baek, et al. designed a real-time PCR assay to amplify the hummingbird specific Avipoxvirus 4b core protein gene (18). Assay development was based on samples from Anna's Hummingbird carcasses, and one *Selasphorus* sp. carcass, all of which had visible pox-like lesions. A data gap exists for establishing assay performance parameters using samples from hummingbirds that appear visually negative for pox and non-Anna's Hummingbird species, which would allow the assay to be used in other bird species.

Many questions remain about the strain of avian pox that infects hummingbirds, including the current prevalence of the virus, the species infected, and the effect of factors such as age, sex, season, and locality on avian pox infections. Determining these baseline characteristics of avian pox in hummingbirds is a crucial first step to address the effects of the virus on hummingbird health and ecosystem services, especially in the context of recent hummingbird population declines (21). It was aimed to answer these questions through a three-pronged approach, examining avian pox prevalence in hummingbirds of California from (1) traditional visual inspection methods with a long-term banding data set, (2) qPCR methods with hummingbird carcasses from multiple species, and (3) qPCR methods with samples collected from live hummingbirds caught in Hall feeder traps.

Banding data were collected from 19 California sites over 18 years, from 2003 to 2020 to model the prevalence of avian pox in six hummingbird species encountered at our banding sites: Black-chinned, Anna's, Costa's (*Calypte costae*), Allen's, Calliope (*Selasphorus calliope*), and Rufous Hummingbirds (*Selasphorus rufus*). In addition, samples were tested from Anna's Hummingbird carcasses without visible pox-like lesions and from carcasses from three additional non-Anna's Hummingbird species, including Allen's Hummingbirds, Black-chinned Hummingbirds, and Ruby-throated Hummingbirds (*Archilochus colubris*). Samples were tested for the presence of *Avipoxvirus* DNA *via* real-time PCR to confirm sensitivity of the assay and determine the optimal sample type for detecting avian pox DNA. These samples, in addition to the samples from carcasses with visual pox-like lesions from Baek, et al. (18), were used to determine the prevalence of avian pox in hummingbird carcasses. Contour feather and blood samples collected in the field from Anna's, Black-chinned, and Allen's Hummingbirds at three different sites in California were used to determine the current prevalence of avian pox in hummingbirds, address the impact of other factors on avian pox infections, and to compare the prevalence of avian pox DNA presence in blood and feather samples.

## Materials and methods

Approval was received from the UC Davis Institutional Animal Care and use Committee (Protocol #22134) to conduct all research within the scope of this study. In addition, approval for bird trapping, handling, and sample collection was obtained from the United States Fish and Wildlife Service (Tell Permit: MB55944B-2), United States Geological Survey Bird Banding Laboratory (Tell Permit: 23947), California Department of Fish and Wildlife (Tell Permit: SC-013066).

### Traditional methods: Banding study

The prevalence of pox-like lesions among hummingbirds in California, USA was quantified using banding data collected from 19 banding sites over 18 years (2003 to 2020). During this period, 13,542 hummingbirds from six species and three genera were banded: Black-chinned, Anna's, Costa's, Allen's, Calliope, and Rufous Hummingbirds (Supplementary Table S1). Birds were identified as potentially infected based on the presence of dry and firm scabbed lesions (pink to yellow in color) that were located at the base of the bill, wings, or legs (20) or around the eyes. Banding sites were classified as either Northern or Southern California depending on whether the site was located north of the 36th parallel. Sites were located on private residences and University of California campuses at Davis and Santa Cruz. Elevations ranged from 6 m to 636 m above sea level. The 17 Northern California sites were located within a 150 km great-circle distance of central coordinates 37.39481° N and −121.3104° W. Both Southern California sites were located within a great-circle distance of 45 km to central coordinates 33.71771° N and −118.3743° W.

The prevalence of avian pox in birds was modeled at the study sites with a generalized linear mixed-effects model using the lme4 package in R (22, 23). A random term for site effects was included in all models to account for variation in prevalence related to environmental factors. No marked individual was ever recaptured after contracting avian pox or recovering from avian pox; therefore, the health status of all birds on first encounter (avian pox/no avian pox) was modeled as an indicator of prevalence in the community. Candidate models were tested including terms for age, sex, genus, and locality and report on the model with the highest AICc weight. AIC values corrected for small samples (**AIC**_**c**_), and AICc weights (**AIC**_**w**_) were calculated using the R package MuMIn version 1.43.17. Frequentist 95% confidence intervals (CI) for generalized linear model beta terms were calculated using the broom package version 0.7.5 (24). Data are presented as mean ± standard error (SE).

### Non-traditional methods: qPCR assay

For the carcass portion of this study, 1,756 samples from 516 hummingbird carcasses were collected between July 2007 and November 2020 from the three species with available study skins. For Anna's Hummingbird carcasses (*n* = 279), 1,120 samples were collected. Samples collected included tail and contour feathers, swabs of lesions, swabs of non-lesioned epithelial regions (beak, periorbital region, and foot), toenail clippings, blood, and tissue from pectoral muscle and from non-feathered epithelial regions, the beak and periorbital region. For Allen's (*n* = 129), Black-chinned (*n* = 52), and Ruby-throated (*n* = 57) Hummingbird carcasses, contour feathers, swabs of the beak region, and toenail clippings were taken (*n* = 634) after this combination of sample types was determined to be optimal. Most carcasses (*n* = 748) were collected from rehabilitation centers where birds died or were submitted by the public after being found dead. A few samples were taken from carcasses that were live birds (*n* = 8) that were then euthanized after being considered unfit to survive due to heavy avian pox infections. Samples were collected following the methods from Baek, et al. (18) and stored at –80 °C until DNA extraction.

For the live-caught bird portion of this study, 1,564 samples from 1,049 hummingbirds were collected between May 2018 and November 2020. Hummingbirds were captured from three sites, one in Northern California, Winters (38.52975°N, 121.8355°W), and two in Southern California, Beverly Hills (34.0961390°N, −118.417814°W) and a private residence in San Diego. Allen's Hummingbird (*n* = 217) samples were only collected from Beverly Hills, and Black-chinned Hummingbird (*n* = 160) samples were only collected from Winters due to insufficient sample size at the other sites. Blood samples were collected for all three species by clipping the distal portion of the bird's toenail and placing the blood either on Whatman FTA (Flinders Technology Associates) cards (GE Healthcare, Chicago, Illinois, USA) or in lysis buffer, which was then stored at –80 °C until DNA extraction (32). Contour feathers were also collected by removing with tweezers contour feathers from each of the four quadrants on the ventral aspect of the bird and stored in paper envelopes at –80 °C until DNA extraction. Carcasses and live hummingbirds caught in the field were both inspected visually for presence of pox-like lesions.

DNA was extracted from each sample using the Wizard Genomics DNA Purification Kit (Promega Corp., Madison, Wisconsin, USA) following methods from Baek et al. (18). Extracted DNA samples were analyzed using either the Qubit 2.0 Fluorometer or the Quant-iT ds DNA High Sensitivity Assay on the BioTek Synergy HT plate reader to ensure successful extraction of DNA. A minimum DNA concentration of 0.5 ng/mL was required for samples to be chosen for further analysis. A total of 25 carcass samples and 16 live bird samples were removed from further analysis due to low DNA concentrations.

Extracted DNA was tested via real-time PCR for amplification of the *Avipoxvirus* 4b core protein gene following methods from Baek et al. (18). Two primers (vAAPV-124f ACGTCAACTCATGACTGGCAAT and vAAPV-246r TCTCATAACTCGAATAAGATCTTGTATCG) and an internal hydrolysis probe

(vAAPV-159*p-*FAM-AGACGCAGACGCTATA-MGB, 5' end, reporter dye FAM [6-carboxyfluorescein], 3' end, quencher dye NFQMGB [Non-Fluorescent Quencher Minor Grove Binding]) were used. Seven μL of a commercially available PCR master mix (TaqMan Fast Advanced Master Mix, Thermo Fisher Scientific, Carlsbad, California, USA, cat #4444557), and 5 μL of extracted DNA were used. One no-template control of purified water was run with each assay, as well as one positive control (AAPV plasmid or known visually positive sample, confirmed by qPCR). Reactions were run on either the CFX 96 Touch Real-Time PCR Detection System (Bio-Rad, Hercules, California, USA) or the StepOnePlus™ Real-Time PCR System (Applied Biosystems, Waltham, Massachusetts, USA) using the following protocol: 50° C for 2 min, 95° C for 10 min, 40 cycles of 95° C for 15 s and 60° C for 1 min. Each 96 well plate contained one positive and one negative control, and 94 samples. The number of cycles required for the florescence to exceed the background fluorescence, the Cq value, was extracted for each sample, with a lower Cq value indicating a higher viral load. In this study, a sample was considered positive for avipoxvirus DNA if the Cq value ≤ 35. Baek et al. (18) used a Cq value cutoff of 40 for a positive sample, where 35–40 was considered a low viral load.

Species-wise avian pox prevalence for sample types from live birds and carcasses was calculated to identify the differences in avian pox detection in various samples. Univariate chi square tests with Yates's adjustment for multiple comparison were used to statistically compare species and sample-wise differences in the detection of avian pox (25). Cq value distributions were compared for blood and feather samples from live birds that had either blood, feather, or both sample types test positive for *Avipoxvirus* DNA. Wilcoxon signed-rank Test was used to compare these two distributions. For samples collected from carcasses sensitivity, negative predictive value, and positive and negative agreements were calculated based on results for all sample types tested for an individual bird, and all three variables take ranges from 0 to 1 (26).

## Results

### Traditional methods: Banding study

**Figure 1:**
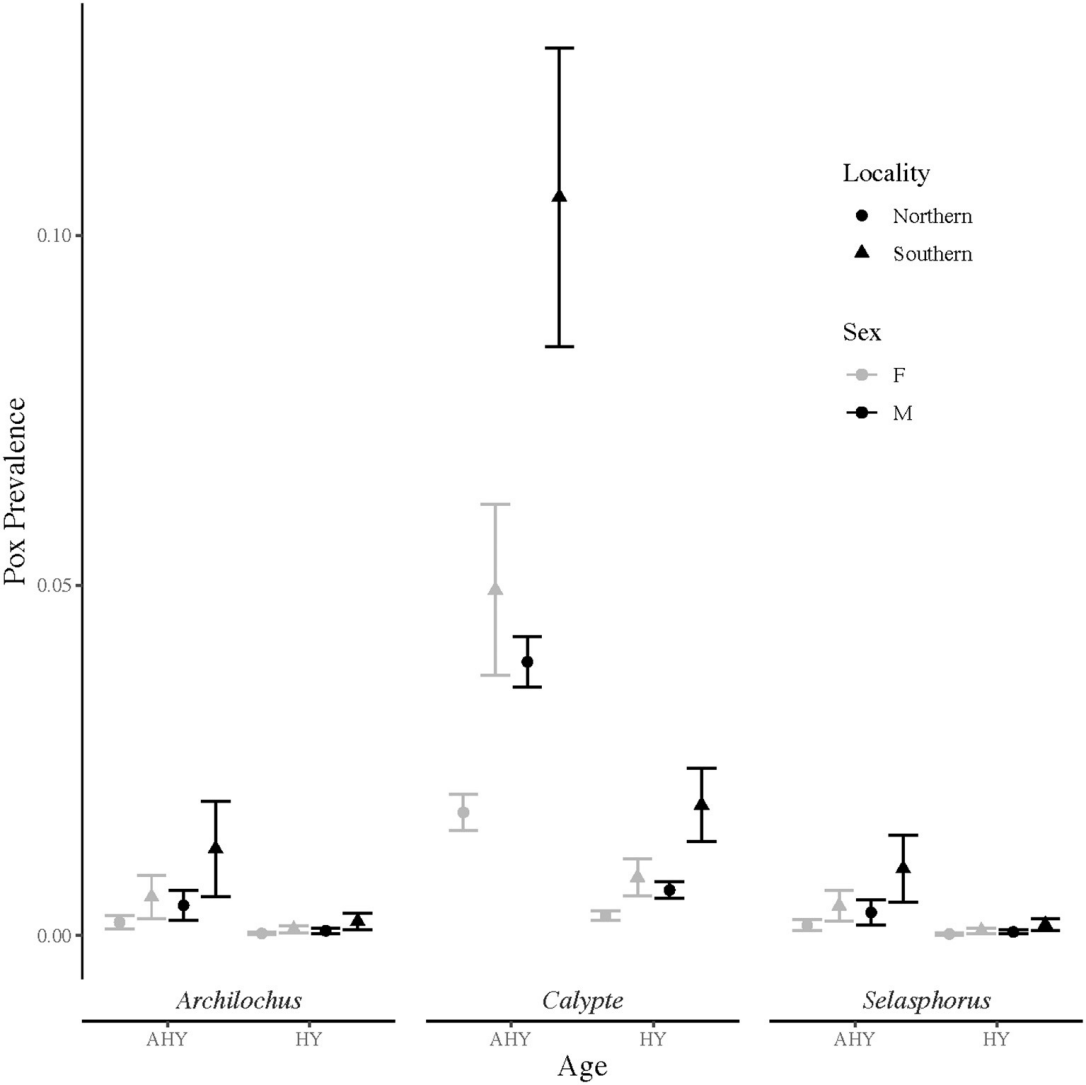
Avian pox prevalence among California hummingbirds based on visual presence of pox-like lesions. Prevalence data were collected from Northern (*n* = 17) and Southern *(n* = 2) California banding sites over 18 years (2003 to 2020). Upper and lower vertical bars indicate prevalence point estimates plus and minus one standard error. Prevalence estimates are shown for after hatch-year (AHY) and hatch-year (HY) birds in the *Archilochus* genus, the *Calypte* genus, and the *Selasphorus* genus.

From 13,542 hummingbird banding records, 196 birds, or about 1.5%, were identified with grossly consistent pox-like lesions. The top model of avian pox prevalence in hummingbirds included terms for age, sex, genus, and locality (Table 1). The *Archilochus* genus comprised Black-chinned Hummingbirds only, while the *Calypte* genus included primarily Anna's Hummingbirds and a small number of Costa's Hummingbirds (10 individuals across all years and sites). The *Selasphorus* genus included Allen's, Rufous, and Calliope Hummingbirds (Supplementary Table S1). Hatch-year birds had significantly lower prevalence of avian pox compared to after hatch-year individuals (β = −1.83 ± 0.2; *z*-value = −9.1; *p-*value ≤ 0.001), and avian pox was significantly more prevalent among males than females (β = 0.82 ± 0.17; z-value = 4.8; *p-*value ≤ 0.001). Avian pox prevalence was significantly higher among Anna's Hummingbirds (the *Calypte* genus) than Black-chinned Hummingbirds (β = 2.2 ± 0.5; *z*-value = 4.4; *p-*value ≤ 0.001). No significant estimates could be derived for the *Selasphorus* genus (β = −0.26 ± 0.73; z-value = −0.36; *p-*value =0.72). Avian pox was significantly more prevalent in the Southern California banding sites compared with the Northern California banding sites, (Figure 1), though our Southern California locality comprised only two sampling sites (β = 1.1 ± 0.2; z-value = 4.5; *p-*value ≤ 0.001).

**Table 1:** Pox prevalence among California hummingbirds.

							**95% CI**		
**Model**	**AIC_c_**	**ΔAIC_c_**	**AIC_w_**	**Term**	**Estimate**	**SE**	**Lower**	**Upper**	***p-*value**
Age +	1,840	0.0	1.0	(Intercept)	−6.26	0.51	−7.45	−5.39	<0.001
Sex +				HY	−1.83	0.20	−2.24	−1.45	<0.001
Genus +				Male	0.82	0.17	0.50	1.16	<0.001
Locality				*Calypte*	2.24	0.51	1.38	3.42	<0.001
				*Selasphorus*	−0.26	0.73	−1.74	1.21	0.72
				Southern	1.06	0.23	0.58	1.50	<0.001
Age +	1,854	14.5	0.0	(Intercept)	−6.27	0.51	−7.46	−5.39	<0.001
Sex +				HY	−1.86	0.20	−2.27	−1.49	<0.001
Genus				Male	0.84	0.17	0.52	1.18	<0.001
				*Calypte*	2.31	0.51	1.45	3.49	<0.001
				*Selasphorus*	0.34	0.71	−1.11	1.78	0.63
Age +	1,864	24.2	0.0	(Intercept)	−5.83	0.50	−7.00	−4.98	<0.001
Genus +				HY	−1.76	0.20	−2.17	−1.38	<0.001
Locality				*Calypte*	2.35	0.51	1.49	3.53	<0.001
				*Selasphorus*	−0.27	0.72	−1.75	1.20	0.71
				Southern	1.11	0.23	0.63	1.55	<0.001
Sex +	1,918	78.4	0.0	(Intercept)	−4.40	0.15	−4.71	−4.12	<0.001
Age +				Male	0.99	0.17	0.66	1.33	<0.001
Locality				HY	−1.75	0.20	−2.16	−1.37	<0.001
				Southern	0.47	0.22	0.01	0.89	0.03
Genus +	1,954	113.9	0.0	(Intercept)	−6.48	0.51	−7.67	−5.61	<0.001
Sex +				*Calypte*	1.96	0.51	1.10	3.14	<0.001
Locality				*Selasphorus*	−0.54	0.72	−2.01	0.94	0.46
				Male	0.67	0.17	0.35	1.01	<0.001
				Southern	1.28	0.23	0.80	1.72	<0.001
Age +	1,955	115.3	0.0	(Intercept)	−3.77	0.08	−3.94	−3.61	<0.001
Locality				HY	−1.63	0.20	−2.04	−1.26	<0.001
				Southern	0.50	0.22	0.04	0.92	0.02
Genus +	1,969	129.1	0.0	(Intercept)	−6.11	0.50	−7.28	−5.27	<0.001
Locality				*Calypte*	2.05	0.51	1.19	3.23	<0.001
				*Selasphorus*	−0.52	0.72	−2.00	0.95	0.47
				Southern	1.35	0.23	0.87	1.78	<0.001
Sex +	2,021	181.7	0.0	(Intercept)	−4.82	0.15	−5.13	−4.54	<0.001
Locality				Male	0.80	0.17	0.48	1.14	<0.001
				Southern	0.66	0.22	0.21	1.08	<0.001
Locality	2,045	205.0	0.0	(Intercept)	−4.29	0.08	−4.44	−4.14	<0.001
				Southern	0.71	0.22	0.25	1.12	<0.001

Prevalence data were collected from 17 Northern banding sites and two Southern banding sites over 18 years (2003 to 2020). Prevalence estimates, standard errors (SE), and 95% confidence intervals (CI) are shown for male and female (reference level) after-hatch-year (AHY) and hatch-year (HY) birds in the *Archilochus genus, the Calypte genus, and the Selasphorus genus*.

### Non-traditional methods: Carcass study

**Figure 2:**
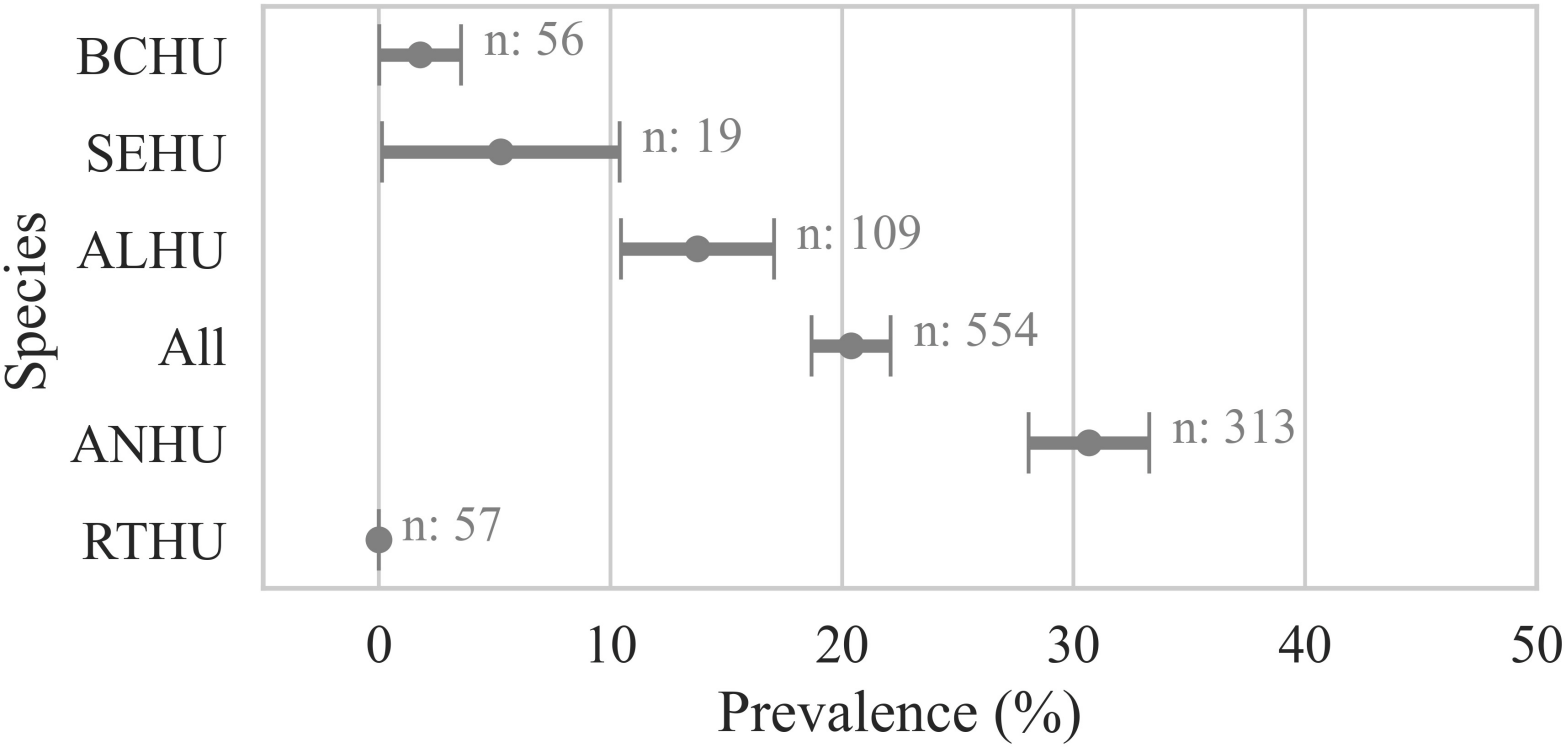
Proportion of hummingbird carcasses by species that were detected positive for avian pox viral DNA. A bird was considered positive (Cq ≤ 35) if any sample from the bird carcass was detected positive for *Avipoxvirus* DNA using a quantitative polymerse chain reaction assay. Points show the prevalence and error bars represent standard error of the mean. ANHU, Anna's hummingbird; BCHU, Black-chinned Hummingbird; ALHU, Allen's Hummingbird, SEHU, *Selasphorus* spp. hummingbird; RTHU, Ruby-throated Hummingbird.

Within individual hummingbird carcasses tested for *Avipoxvirus* DNA using various samples, 20 ± 2% (*n* = 554) were positive for at least one sample. Anna's Hummingbird carcasses showed significantly higher avian pox prevalence (30 ± 3%, *n* = 313, χ2 *p-*value < 0.001) compared to Allen's Hummingbird carcasses (13 ± 4%, *n* = 109), *Selasphorus* spp. hummingbirds (5 ± 6%, *n* = 19), and Black-chinned Hummingbirds (1 ± 2%, *n* = 56, Figure 2).

All pox-like lesion samples that were collected from carcasses were positive for *Avipoxvirus* DNA with the qPCR test (*n* = 33). Besides lesions, tissues collected from carcasses that were visually negative showed significantly higher (χ2 *p-*value < 0.001) prevalence of 42 ± 5% (*n* = 89) than other sample types (feathers and non-lesion swabs). Within feathers sampled from carcasses of Anna's Hummingbirds, the prevalence was significantly higher (χ2 *p-*value < 0.005) for body feathers (44 ± 7%, *n* = 57) than rectrices (13 ± 2%, *n* = 228). Results for swabs taken from anatomic locations where pox lesions commonly occur but no lesions were present were as follows: beak (23 ± 6%, *n* = 53), toe (31 ± 6%, *n* = 64), and peri-orbital regions (36 ± 6%, *n* = 66) and did not show any significant differences (χ2 *p-*value = 0.61) in the prevalence of pox for Anna's Hummingbird carcasses (Table 2). Table 2 shows proportions of sub-sample types that tested positive for avian pox DNA from live hummingbirds and carcasses.

**Table 2:** Proportion of sample types and sub-sample types identified positive for avian poxvirus DNA from live and carcasses of various hummingbird species caught or found in California.

**Bird status**	**Hummingbird**	**Sample type**	**Sub-sample**	**Number of Positive samples**	**Prevalence**	**Standard error**	***n* (birds)**
	**species**		**identification**				
Live	ALHU	Feather	Body	25	0.13	0.02	192
	ANHU	Blood	FTA	15	0.11	0.03	133
		Blood	Lysis buffer	9	0.02	0.01	379
		Feather	Body	385	0.6	0.02	646
	BCHU	Feather	Body	24	0.14	0.03	167
Carcass	ALHU	Feather	Body	10	0.11	0.03	90
		Non-lesion swab	Beak	5	0.06	0.02	90
	ANHU	Blood	Nobuto	1	1	0	1
		Blood	FTA	3	0.5	0.2	6
		Feather	Wing	2	1	0	2
		Feather	Body	25	0.44	0.07	57
		Feather	Tail	29	0.13	0.02	228
		Lesion swab	Eye	5	1	0	5
		Lesion swab	Keel	1	1	0	1
		Lesion swab	Leg	1	1	0	1
		Lesion swab	Toe	16	1	0	16
		Lesion swab	Wing	7	1	0	7
		Lesion swab	Beak	19	0.95	0.05	20
		Non-lesion swab	Eye	24	0.36	0.06	66
		Non-lesion swab	Toe	20	0.31	0.06	64
		Non-lesion swab	Beak	12	0.23	0.06	53
		Tissue	Keel	2	1	0	2
		Tissue	Wing	5	0.83	0.15	6
		Tissue	Beak	19	0.29	0.06	65
		Tissue	Muscle	19	0.27	0.05	70
		Tissue	Eye	19	0.27	0.05	71
		Toenail	Toe	35	0.15	0.02	234
	BCHU	Feather	Tail	1	1	0	1
	SEHU	Lesion swab	Toe	1	1	0	1
		Lesion swab	Muscle	1	1	0	1
		Non-lesion swab	Beak	1	1	0	1
		Non-lesion swab	Toe	1	1	0	1
		Toenail	Toe	1	0.06	0.05	18

*Molecular testing was performed using a quantitative polymerase chain reaction assay*.

*ANHU, Anna's Hummingbird; BCHU, Black-chinned Hummingbird; SEHU, Selasphorus spp. hummingbird; FTA, Whatman Flinders Technology Associates cards*.

Body feathers and rectrix samples from Anna's Hummingbird carcasses together showed a sensitivity of 0.73 with positive agreement of 0.845 and negative agreement of 0.96 with true status of the bird. Within feather sample types, body feathers showed higher sensitivity of 0.67 compared to rectrices (0.60). All birds sampled with pox-like lesions were also positive by the qPCR assay for *Avipoxvirus* DNA, but lesion tissue samples from the beak showed sensitivity of 0.95 (*n* = 20). Swabs taken from anatomic locations where pox lesions commonly occur, but no lesions were present (non-lesion swabs) for Anna's Hummingbird carcasses showed sensitivity of 0.89 with high agreement with true status of the bird (negative agreement = 0.94 and positive agreement 0.94). Tissue samples without pox-like lesions from Anna's Hummingbird carcasses showed sensitivity of 0.69 with moderately high agreement with true status of the bird (negative agreement = 0.81 and positive agreement = 0.81). Sensitivity for individual lesion tissue sample types was lower than their combined sensitivity, with beak, eye, and muscle tissues showing sensitivities of 0.57, 0.48 and 0.48 respectively. Toenail samples Anna's Hummingbird carcasses had a sensitivity of 0.64 with positive agreement of 0.78 and a high negative agreement of 0.90 with the true status of the individual bird.

**Figure 3:**
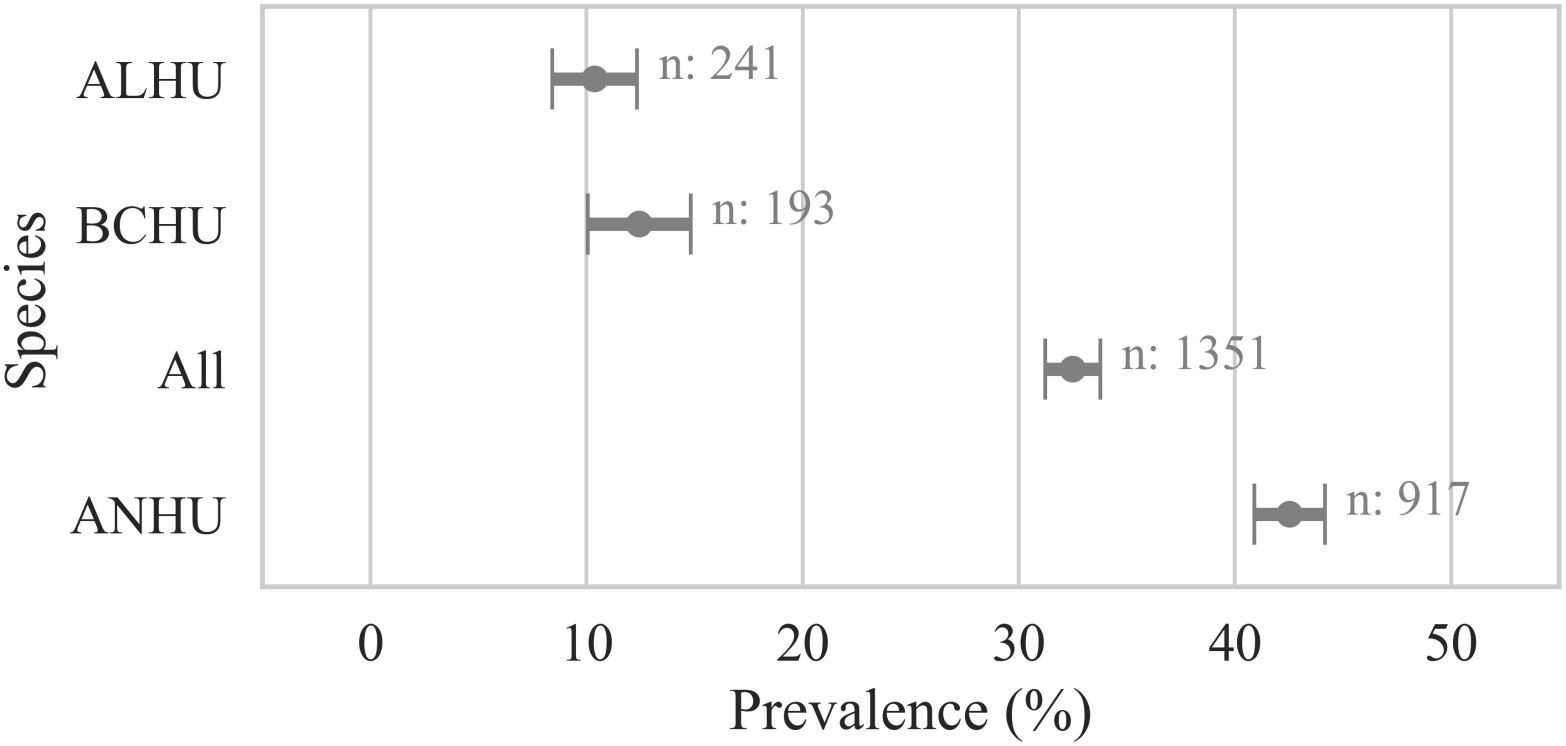
Proportion of live birds by species that were detected positive for *Avipoxvirus* DNA. A bird was considered positive (Cq ≤ 35) if any sample from the bird was detected positive for *Avipoxvirus* DNA using a quantitative polymerse chain reaction assay. Points show the prevalence and error bars represent standard error of the mean. ANHU, Anna's Hummingbird; BCHU, Black-chinned Hummingbird; ALHU, Allen's Hummingbird.

When explored sample-wise, feather samples and non-lesion swab samples from carcasses that had pox-like lesions did not show significantly lower Cq values, the number of cycles required for fluorescence to exceed the background signal, compared to birds that did not have pox-like lesions (Mann Whitney U *P-*value < 0.05). Cq values for tissue and toenail samples did not show a significant difference between birds that visually had pox-like lesions vs. bird that visually did not have pox-like lesions (Figure 3). Male carcasses showed higher prevalence overall (24 ± 3%, *n* = 253) compared to female carcasses (20 ± 3%, *n* = 213). Carcasses collected from Northern California showed significantly lower prevalence (6 ± 2%, *n* = 138) than carcasses collected from Southern California (38 ± 3%, *n* = 215, χ2 *p-*value < 0.001). The prevalence of *Avipoxvirus* DNA in hatch-year carcasses (12 ± 3%, *n* = 217) was significantly lower than prevalence detected in after hatch-year carcasses (31 ± 3%, *n* = 236, χ2 *p-*value < 0.001).

### Non-traditional methods: Live bird study

**Figure 4:**
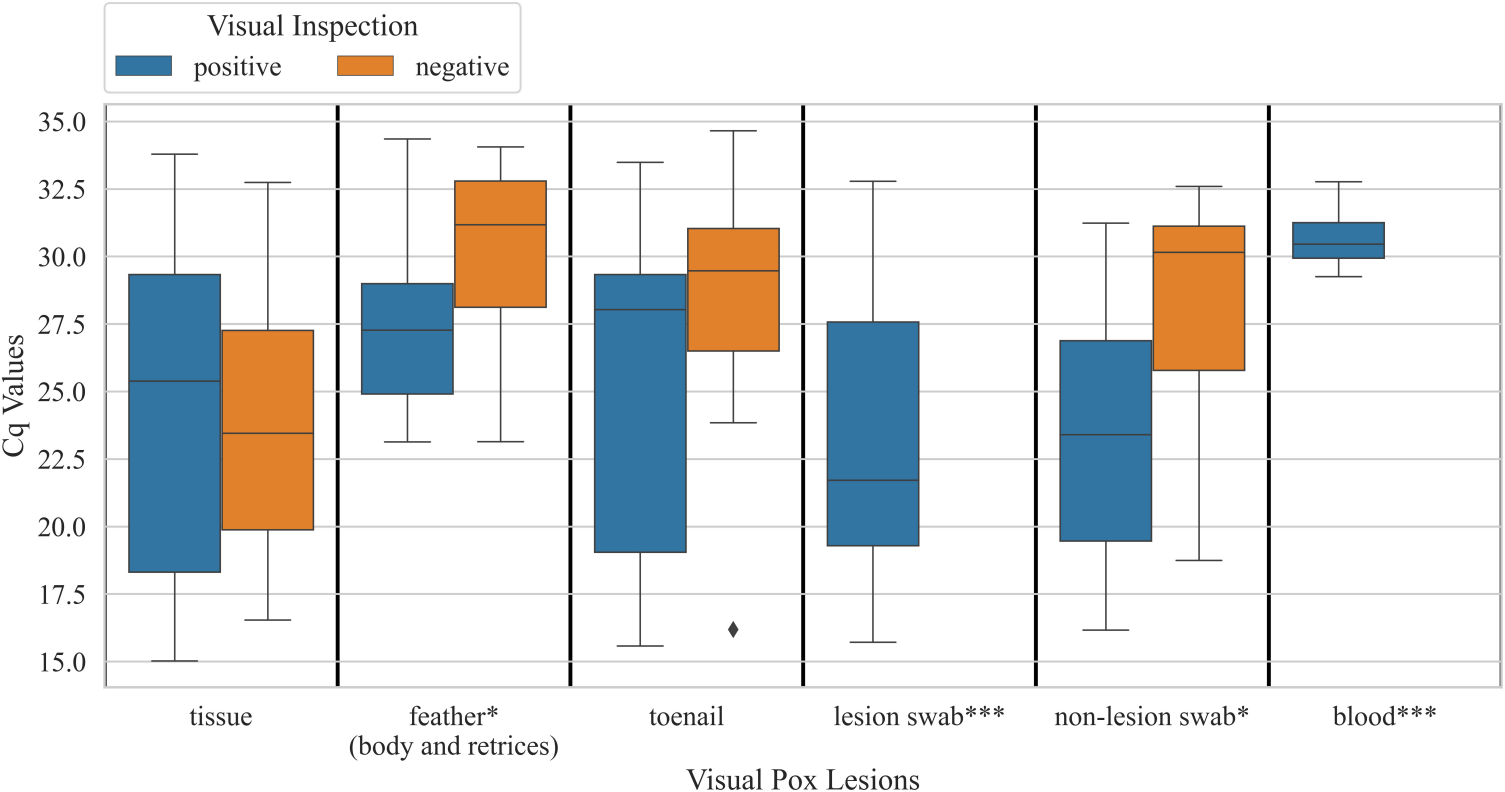
Boxplots showing distribution of Cq values for various sample types obtained from hummingbird carcasses that were visually positive for pox-like lesions vs. carcasses that were visually negative for pox-like lesions (* Mann Whitney U *P-*value <0.05; ***, visually negative birds not observed). Samples were tested for *Avipoxvirus* DNA using a quantitative polymerase chain reaction assay.

Samples collected from live-caught hummingbirds showed avian pox prevalence of 32 ± 2% (*n* = 1,351) at the individual bird level. Similar to results from carcasses, samples from live Anna's Hummingbirds showed significantly higher prevalence (42 ± 2%, *n* = 917, χ2 *p-*value < 0.005) than other species of hummingbirds sampled (Figure 4).

**Figure 5:**
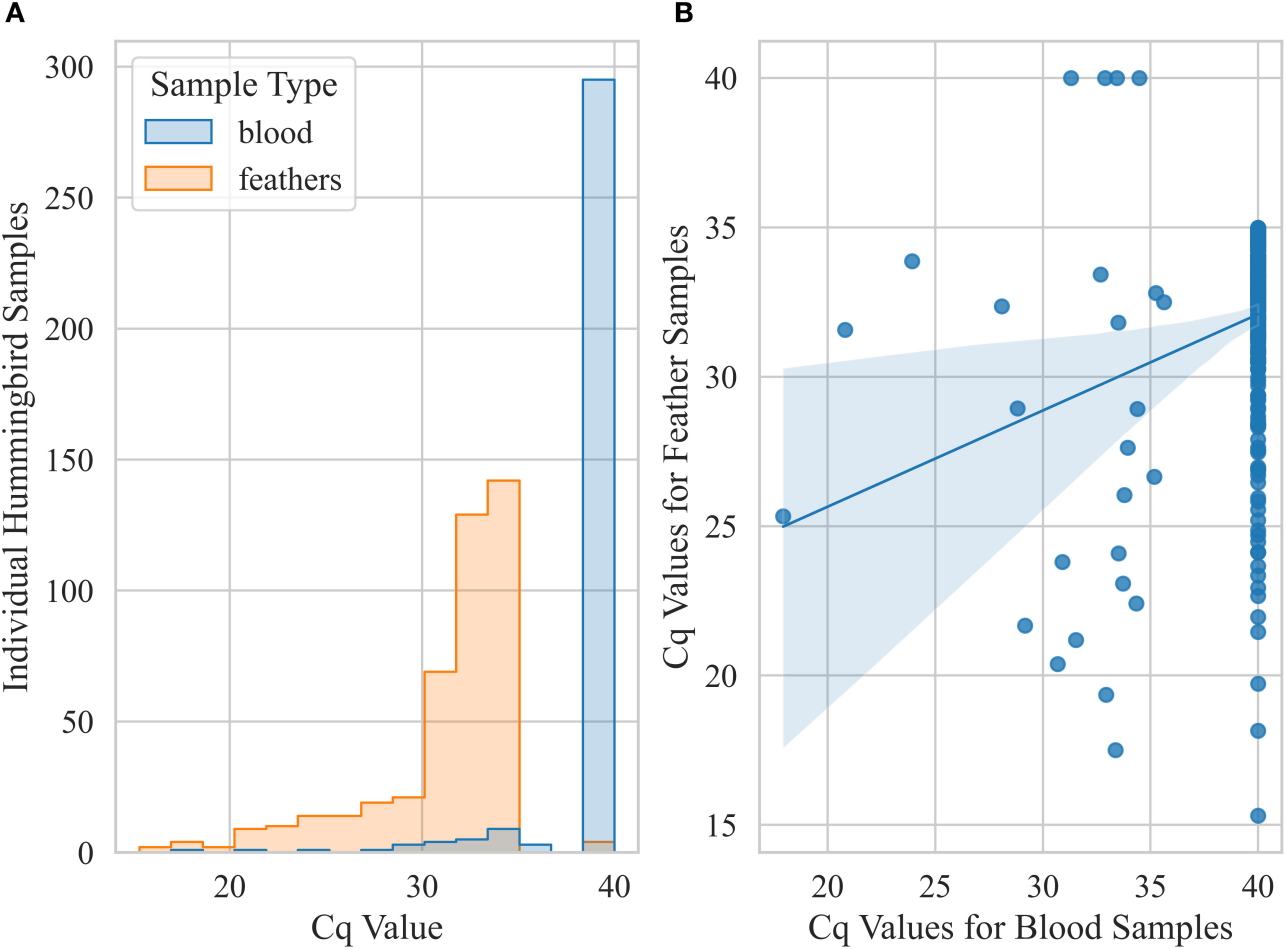
Pairwise comparison of Cq values for blood and feather samples from live hummingbirds caught in California that were classified positive for at least one of these two sample types. A bird was considered positive (Cq ≤ 35) if any sample was detected positive for *Avipoxvirus* DNA using a quantitative polymerase chain reaction assay. **(A)** histogram showing distributions of Cq values for blood and feather (body and retrices) samples **(B)** scatter plot showing correlation between blood and feather qPCR Cq values.

Prevalence based on only feather samples from live-caught birds was 43 ± 2% (*n* = 1,014) but reduced to 3 ± 1% (*n* = 893) based on only blood samples. Within live birds sampled, body feather samples showed significantly higher prevalence (χ2 *p-*value < 0.001) for Anna's Hummingbirds (59 ± 2%, *n* = 646) compared to Black-chinned Hummingbirds and (14 ± 3%, *n* = 167) and Allen's Hummingbirds (13 ± 3%, *n* = 192). Pairwise comparison of Cq values for blood and feather samples from live birds that were detected positive for *Avipoxvirus* DNA for at least one of these two samples showed that blood samples had significantly lower Cq values compared to feather samples from the same individual birds (paired *t*-test, *p-*value < 0.005, Figure 5). Live positive birds (positive for either blood or feather) that had visual pox-like lesions had significantly lower Cq values compared to qPCR positive for *Avipoxvirus* DNA birds that had no visual pox-like lesions (Mann-Whitney U *P-*value < 0.005). Males showed higher prevalence (positive for either blood or feather) (45 ± 3%, *n* = 562) compared to females (38 ± 3%, *n* = 488). Similar to prevalence estimates based on hummingbird carcasses and visual inspection during banding operations, live birds captured in Northern California reflected significantly lower prevalence (26 ± 2%, *n* = 417) than live birds captured in Southern California (52 ± 2%, *n* = 638) based on their positivity for either of the sample type (blood or feather). Finally, the prevalence (positive blood or feather sample) was statistically similar across live hatch-year (40 ± 3%, *n* = 323) and after hatch-year birds (42 ± 2%, *n* = 582, *p* = 0.72).

## Discussion

This is the first study that supports the use of non-invasive collection of integument samples using highly sensitive qPCR molecular testing on a large-scale that could change the landscape of diagnosing avian pox in other free-ranging species. This study also is the first to determine the prevalence of avian pox in members of the Family *Trochilidae*, an ecologically important and diverse family of birds. Avian pox prevalence in hummingbird species ranged from 1.5% from banding data, 20.36% from hummingbird carcasses, and 32.47% from live hummingbirds. These prevalences of presence of avian pox virus DNA based on our qPCR assay are higher than expected and show how traditional visual methods of estimating avian pox prevalence can grossly underestimate the population prevalence as predicted by molecular testing.

Traditional prevalence studies on avian pox have used either visual inspection of birds for pox-like lesions or histologic analysis of lesions to confirm the presence of characteristic Bollinger bodies. These methods can vastly underestimate the prevalence of avian pox, as many infections can be missed due to lesion sloughing or asymptomatic birds that do not have visually detectable lesions. Banders handling birds can also easily miss small lesions or not properly examine the whole bird for presence of lesions, again leading to an underestimate of avian pox prevalence. Baek et al. developed a new method for detecting avian pox DNA in hummingbird samples collected non-invasively using a real-time PCR assay. Samples, such as feathers, toenails, and swabs, allowed for greater accuracy in avian pox detection, although this was only tested on Anna's Hummingbirds that had visible pox-like lesions (18). This original study that looked at sensitivity was expanded to identify the ability to diagnose a positive case, and added specificity, the ability to diagnose a bird negative for avian pox based on no visual signs of lesions, using samples from hummingbird carcasses without pox-like lesions and from four additional hummingbird species.

PCR results for swabs from pox susceptible locations but with no lesions revealed that peri-orbital, beak, and toenail regions had the highest sensitivity, while feather (body or rectrices) samples also exhibited moderately high sensitivity. A combination of swabs taken from non-lesion anatomic regions where avian pox-lesions are commonly found and body feathers appeared to be the most sensitive and had the highest sensitivity for detecting avian pox infections using this qPCR assay. Although epithelial samples are not commonly used for avian pox detection in live birds, these findings demonstrate their utility for avian pox monitoring in wild birds (18).

Hummingbird carcasses, although important in addressing initial questions about avian pox in hummingbirds, are limited in scope of results relative to random sampling from a live bird population, due to possible confounding variables such as the cause of death and the duration birds were held in captivity at the rehabilitation centers. Body feather and blood samples were collected from living hummingbirds to determine the prevalence of avian pox in California hummingbirds. Blood samples had significantly lower Cq values than body feather samples from the same individuals, indicating that infected birds have circulating viral loads in their bloodstream. The additional sensitivity of this qPCR assay may have added to the detection possibility of avian pox from blood samples. Among birds that tested positive *via* qPCR, birds with visible pox-like lesions had significantly lower Cq values, indicating a higher viral load, than birds with no visible pox-like lesions. This indicates that birds with visible pox-like lesions have a more severe avian pox infection or have a more recent infection.

The patterns of prevalence of avian pox in hummingbirds were similar throughout the three methods, although estimated avian pox prevalence was highest using the new qPCR assay. Anna's Hummingbirds harbored a higher prevalence of avian pox than other species in the banding dataset, the carcass study, and in the live bird study. This supports field observations that Anna's Hummingbirds have a higher prevalence of pox-like lesions. This species-level difference in susceptibility to avian pox could be driven by multiple factors, including differences in species immune responses, differences in time spent at feeders or in high vector abundance habitats, differences in vector preferences for Anna's Hummingbirds, or even differences in social interactions between species. Although all of these factors are unknown, they provide future avenues to explore to address the reason behind this species-level difference.

An individual's probability of becoming infected with a pathogen is dependent on numerous characteristics, including age, sex, health, immune response, location, and season (27). Juvenile birds harbor higher prevalences of avian pox in a wide variety of bird species (14). In this study, after hatch-year hummingbirds showed a higher prevalence of avian pox compared with hatch-year hummingbirds, and this pattern was significant in carcasses but not in live birds. Hatch-year birds may have less relative time exposure, less chances to become infected with avian pox, and less seasonal exposure compared to adults for when the vector is active, and the virus is circulating.

Host sex does not affect avian pox virus infection propensity in most species (14). However, male hummingbirds in this study showed a higher prevalence of avian pox compared with female hummingbirds. In hummingbirds, males and females differ greatly in their interactions with other hummingbirds. Male hummingbirds can become aggressively territorial around feeders, and physically eject other birds from defended resource-rich sites (28, 29). Male hummingbirds may therefore come into direct contact with other infected hummingbirds. Avian pox can be spread through direct contact with infected individuals, suggestive of a mechanism by which male hummingbirds harbor a higher prevalence of avian pox than females, with the caveat that contact transmission would require damage to the integument or the compromise of a mucosal surface for transmission to occur.

Avian pox prevalence can be influenced by the abundance of vectors, which in turn is driven by landscape seasonality and characteristics including elevation, rainfall (14). It was found that hummingbirds from Northern California harbored a lower prevalence of avian pox than hummingbirds from Southern California. Differences in vector population size, due to temperature preferences, humidity preferences, or available habitat between Northern and Southern California could be driving this difference. The Northern California sites on average have a lower monthly average temperature and lower humidity than the Southern California sites, which could provide better conditions for mosquitoes, a common mechanical vector of avian pox.

Hummingbird diversity peaks around the equator in the western hemisphere and diffuses to both the north and south (30). Expanding future studies to more areas and more hummingbird species would be critical to determining the true spread of the virus in hummingbirds, and possibly determining the origin of the virus in hummingbirds. It is unclear whether the virus co-evolved with hummingbirds or more recently spread by cross-species transfer.

One caveat of this study was that all birds were caught using Hall feeder traps. This approach biased the study in favor of hummingbirds that utilize and spend more time in urban environments and utilize hummingbird feeders. It is proposed that future studies aim to assess avian pox prevalence among hummingbirds in non-urbanized regions and not during migration. Prevalence of avian pox is known to increase in populations with anthropogenic food provisioning in the form of feeders (31). Hummingbird feeders may increase avian pox infections, as they create high-density areas of hummingbirds during crepuscular hours, when mosquitoes, the most common insect vector known for transmitting avian pox, are also abundant. Avian pox transmission by contact at feeders is also possible but would require damage to the integument. Additionally, the feeders themselves could be a source of infection, with infected birds ingesting contaminated sugar water, although the birds would have to have a mucosal surface compromised.

Hummingbirds are a charismatic and ecologically important group of birds present all throughout the Americas. Although hummingbirds are easily recognized by humans, the pathogens impacting hummingbird health are relatively unknown. Our research identifies the current prevalence of avian pox in hummingbirds and establishes a method of identifying avian pox positive birds that can be applied to other wild bird species. Our higher-than-expected prevalence of avian pox in hummingbirds may have large implications for hummingbird population health and highlights the need for future focus on the pathogens of wild animals.

## Data availability statement

The original contributions presented in the study are included in the article/Supplementary material, further inquiries can be directed to the corresponding author/s.

## Ethics statement

The animal study was reviewed and approved by UC Davis Institutional Animal Care and Use Committee (Protocol #22134).

## Author contributions

AG, PP, RS, and LT conceived the ideas, designed methodology and led the writing of the manuscript. AG, RQ RB, RC, BR, HE, and MB collected the data. AG, PP, SE, and RB analyzed the data. RS and LT contributed equally to this publication in terms of conception and mentoring for this project. All authors contributed to the article and approved the submitted version.

## Funding

This work was in part supported by a grant to RNMS, NIH 1SC3GM118210-01A.

## Conflict of interest

The authors declare that the research was conducted in the absence of any commercial or financial relationships that could be construed as a potential conflict of interest.

## Publisher's note

All claims expressed in this article are solely those of the authors and do not necessarily represent those of their affiliated organizations, or those of the publisher, the editors and the reviewers. Any product that may be evaluated in this article, or claim that may be made by its manufacturer, is not guaranteed or endorsed by the publisher.
