# Supplementary material for: Evaluation of minimally invasive sampling methods for detecting Avipoxvirus: Hummingbirds as a case example
Source: Front Vet Sci. 2022 Aug 24;9:924854. doi: 10.3389/fvets.2022.924854 (PMC9450938; doi:10.3389/fvets.2022.924854)
Supplement: Supplementary file 1 [file Table_1.pdf]

Supplementary Material

1 Supplementary Tables

Genus	Species	Sex	Age	Locality	n	Pox
Archilochus	BCHU	F	AHY	Northern	719	2
Archilochus	BCHU	F	AHY	Southern	4	0
Archilochus	BCHU	F	HY	Northern	219	0
Archilochus	BCHU	F	HY	Southern	1	0
Archilochus	BCHU	M	AHY	Northern	538	2
Archilochus	BCHU	M	HY	Northern	310	0
Archilochus	BCHU	M	HY	Southern	2	0
Calypte	ANHU	F	AHY	Northern	1974	32
Calypte	ANHU	F	AHY	Southern	84	4
Calypte	ANHU	F	HY	Northern	1819	8
Calypte	ANHU	F	HY	Southern	9	0
Calypte	ANHU	M	AHY	Northern	2681	110
Calypte	ANHU	M	AHY	Southern	142	13
Calypte	ANHU	M	HY	Northern	3362	18
Calypte	ANHU	M	HY	Southern	108	3
Calypte	COHU	F	HY	Northern	1	0
Calypte	COHU	M	AHY	Northern	2	0
Calypte	COHU	M	HY	Northern	7	0
Selasphorus	ALHU	F	AHY	Northern	166	0
Selasphorus	ALHU	F	AHY	Southern	175	1
Selasphorus	ALHU	F	HY	Northern	38	0
Selasphorus	ALHU	F	HY	Southern	25	0
Selasphorus	ALHU	M	AHY	Northern	57	0
Selasphorus	ALHU	M	AHY	Southern	182	2
Selasphorus	ALHU	M	HY	Northern	62	0

Selasphorus	ALHU	M	HY	Southern	137	1
Selasphorus	CAHU	F	AHY	Northern	71	0
Selasphorus	CAHU	F	HY	Northern	1	0
Selasphorus	CAHU	M	AHY	Northern	19	0
Selasphorus	CAHU	M	HY	Northern	2	0
Selasphorus	RUHU	F	AHY	Northern	105	0
Selasphorus	RUHU	F	AHY	Southern	9	0
Selasphorus	RUHU	F	HY	Northern	174	0
Selasphorus	RUHU	M	AHY	Northern	78	0
Selasphorus	RUHU	M	AHY	Southern	2	0
Selasphorus	RUHU	M	HY	Northern	257	0

Supplementary Table 1. Tallied hummingbird encounters in California USA across 18 years (2003 to 2020) and 2 localities. Localities comprised 17 northern sites and 2 southern sites. Species in the *Archilochus* genus include only Black-chinned Hummingbirds (*A. alexandri*; BCHU). The *Calypte* genus includes Anna's (*C. anna*); ANHU) and Costa's (*C. costae*; COHU) Hummingbirds. The *Selasphorus*. genus includes Allen's (*S. sasin*; ALHU), Rufous (*S. rufus*; RUHU), and Calliope (*S. calliope*; CAHU) Hummingbirds.
